# CLCC1 c. 75C>A Mutation in Pakistani Derived Retinitis Pigmentosa Families Likely Originated With a Single Founder Mutation 2,000–5,000 Years Ago

**DOI:** 10.3389/fgene.2022.804924

**Published:** 2022-03-22

**Authors:** Yan Ma, Xun Wang, Nadav Shoshany, Xiaodong Jiao, Adrian Lee, Gregory Ku, Emma L. Baple, James Fasham, Raheela Nadeem, Muhammad Asif Naeem, Sheikh Riazuddin, S. Amer Riazuddin, Andrew H. Crosby, J. Fielding Hejtmancik

**Affiliations:** ^1^ Ophthalmic Genetics and Visual Function Branch, National Eye Institute, Bethesda, MD, United States; ^2^ State Key Laboratory of Ophthalmology, Zhongshan Ophthalmic Center, Sun Yat-sen University, Guangzhou, China; ^3^ Matlow’s Ophthalmo-genetic Laboratory, Shamir Medical Center, Zeriffin, Israel; ^4^ Diabetes Center, University of California, San Francisco, San Francisco, CA, United States; ^5^ Research, Innovation, Learning and Development (RILD) Wellcome Wolfson Centre, College of Medicine and Health, University of Exeter Medical School, Royal Devon and Exeter NHS Foundation Trust, Exeter, United Kingdom; ^6^ Peninsula Clinical Genetics Service, Royal Devon and Exeter Hospital (Heavitree), Gladstone Road, Exeter, United Kingdom; ^7^ National Centre of Excellence in Molecular Biology, University of the Punjab, Lahore, Pakistan; ^8^ Allama Iqbal Medical College, University of Health Sciences, Lahore, Pakistan; ^9^ The Wilmer Eye Institute, Johns Hopkins University School of Medicine, Baltimore, MD, United States

**Keywords:** retinitis pigmenstosa, CLCC1, haplotype analysis, mutation age estimate, Pakistan, founder mutation

## Abstract

**Background:** A CLCC1 c. 75C > A (p.D25E) mutation has been associated with autosomal recessive pigmentosa in patients in and from Pakistan. CLCC1 is ubiquitously expressed, and knockout models of this gene in zebrafish and mice are lethal in the embryonic period, suggesting that possible retinitis pigmentosa mutations in this gene might be limited to those leaving partial activity. In agreement with this hypothesis, the mutation is the only CLCC1 mutation associated with retinitis pigmentosa to date, and all identified patients with this mutation share a common SNP haplotype surrounding the mutation, suggesting a common founder.

**Methods:** SNPs were genotyped by a combination of WGS and Sanger sequencing. The original founder haplotype, and recombination pathways were delineated by examination to minimize recombination events. Mutation age was estimated by four methods including an explicit solution, an iterative approach, a Bayesian approach and an approach based solely on ancestral segment lengths using high density SNP data.

**Results:** All members of each of the nine families studied shared a single autozygous SNP haplotype for the CLCC1 region ranging from approximately 1–3.5 Mb in size. The haplotypes shared by the families could be derived from a single putative ancestral haplotype with at most two recombination events. Based on the haplotype and Gamma analysis, the estimated age of the founding mutation varied from 79 to 196 generations, or approximately 2,000–5,000 years, depending on the markers used in the estimate. The DMLE (Bayesian) estimates ranged from 2,160 generations assuming a population growth rate of 0–309 generations assuming a population growth rate of 2% with broad 95% confidence intervals.

**Conclusion:** These results provide insight into the origin of the CLCC1 mutation in the Pakistan population. This mutation is estimated to have occurred 2000–5,000 years ago and has been transmitted to affected families of Pakistani origin in geographically dispersed locations around the world. This is the only mutation in CLCC1 identified to date, suggesting that the CLCC1 gene is under a high degree of constraint, probably imposed by functional requirements for this gene during embryonic development.

## Introduction

Retinitis pigmentosa (RP [MIM 268000]) is a clinically and genetically heterogeneous disorder affecting approximately one in 4,000 individuals worldwide (Hartong et al., 2006). Clinically, patients initially exhibit night blindness followed by progressive loss of peripheral visual fields, eventually culminating in compromise or even complete loss of central vision. Typical fundus changes include bone spicule-like pigmentation in the mid-peripheral retina, waxy pallor of the optic discs, and attenuation of retinal blood vessels. Since RP initially affects the rod photoreceptors, followed by the degeneration of cone photoreceptors, patients often have severely diminished or extinguished rod response in electroretinography (ERG) even in early stages of the disease, while the cone response is relatively preserved initially but decreases and becomes undetectable as the disease progresses (Bird, 1995). Genetic inheritance patterns of RP include autosomal-dominant (about 30–40% of cases), autosomal-recessive (50–60%), and X-linked (5–15%) inheritance (Bunker et al., 1984; Rivolta et al., 2002). More than 82 causative genes have been identified for RP so far, of which 58 genes have been identified in families with autosomal recessive RP (arRP) (Daiger et al., 2021).

At least in part reflecting social and economic considerations, the frequency of consanguineous marriages in Pakistan is among the highest in the world (Bittles, 2001), ranging from 15 to 35% (Hamamy et al., 2011). In reviewing 146 genetically resolved arRP Pakistani families, Khan et al. found only 4 (2.7%) with compound heterozygous mutations (Khan et al., 2014), emphasizing the role of consanguinity on the incidence of arRP in this population. Not only does the high frequency of consanguinity in the Pakistani population bring out autosomal recessive alleles, but it also increases the likelihood that sharing of variations by different families is likely to be the result of the variant allele being derived from a common ancestor, especially if the families that share the same variation also share a common intragenic SNP haplotype for the associated gene.

Chloride channel CLIC like 1 (CLCC1) is a transmembrane channel protein with high permeability for anions, in particular chloride, localized to the endoplasmic reticulum (ER) and in some cell types possibly the Golgi apparatus and Nucleus (Nagasawa et al., 2001). The *CLCC1* gene spans 33 kb, comprising 13 exons encoding a 551 amino acid protein. Li et al. (Li et al., 2018) demonstrated that Clcc1 is highly expressed in the mouse retina, and modestly expressed in the iris, optic nerve, sclera, and cornea. Immunohistochemistry in the normal adult human eye demonstrated CLCC1 expression extensively in the retina and optic nerve, suggesting a physiologic role of CLCC1 in retinal function. Within the retina, CLCC1 staining was more intense in the lamina cribrosa, optic nerve, ganglion cell layer, inner and outer nuclear layers, and retinal pigment epithelium (RPE). The CLCC1 NM_145,543.2:c.75C > A (p.D25E) missense mutation in *CLCC1* was found in seven Pakistani families and one British-Bangladeshi family with arRP mapping to chromosome 1p13 (RP32; 609,913). Recent additional screening has found one new family (61334) carrying the same mutation, bringing the total number of families to nine and accounting for about 6% of genetic cases of arRP in Pakistani families (Li et al., 2017).

The present study was undertaken to investigate the possible common ancestry of the nine Pakistani and Pakistani-derived families carrying the c.75C > A mutation, to define the likely recombination and mutational events that would be required to occur if they did have a common founder, to estimate the approximate age of the putative founder mutation and to correlate the history and geographic distribution of this mutation with the population history of Pakistan. To achieve these goals, we performed haplotype analysis of 99 intragenic SNPs flanking the c.75C > A CLCC1 mutation, derived the recombinational pathways requiring the fewest recombination events to yield the currently observed haplotypes, and estimated the number of generations that have occurred since the original mutation in the founder.

## Materials and Methods

### Patients and DNA Samples

This study was approved by the Institutional Review Boards (IRB) of the National Centre of Excellence in Molecular Biology, Lahore, Pakistan, and the CNS IRB at the National Institutes of Health, and consent was obtained in accordance with the Declaration of Helsinki. Patients were diagnosed with RP on the basis of clinical features as previously described (Li et al., 2018). Blood samples were collected from potentially informative family members, and genomic DNA was extracted from leukocytes according to standard protocols (Smith et al., 1989).

Nine retinitis pigmentosa families carrying the c.75C > A mutation were identified in Pakistan originating from the Pakistani (Punjab) origin. Eight families (FAM1, FAM2, FAM3, 61030, 61031, 61224, 61244 and 61328) have been reported previously (Li et al., 2018). Family 61334 is a newly reported consanguineous Pakistani family with non-syndromic RP carrying the same mutation.

### SNP Marker Analysis

For Families one to three SNP genotyping was performed using Illumina Human CytoSNP-12v2.1 330K arrays. For the remaining families 99 SNPs in a 3.5 Mb centered on the CLCC1 NM_145,543.2 c.75C > A (NC_000001.11:g. 108950376G > T) mutation, were genotyped (Table 1). PCR amplification and analysis of the 99 SNPs sequence was performed as reported previously (Li et al., 2018). PCR products were purified using Agencourt CleanSEQ (Beckman Coulter, Biomek, Brea, CA, United States), sequenced on an ABI PRISM 3130 Automated sequencer (Applied Biosystems, Foster City, CA, United States) and analyzed using Mutation Surveyor v5.1.1 (Soft Genetics, State College, PA, United States) or Lasergene 16.0 (DNASTAR, Madison, WI, United States). In addition, WGS was performed on families 61244, 61334, and 61030.

**TABLE 1:** Summary of the haplotypes for each family. In Table 1 in the Supplementary Material the original risk haplotype is shown in blue and recombined haplotypes held in common by pairs of families showing identical recombination points are highlighted various shades of green. The haplotype blocks composed of SNPs showing no recombination in affected members of the nine families are shown in alternating orange and yellow in the marker column (GRCh38.p13). The genomic position of the CLCC1 mutation is shown in bold type.

Position (GRCh38)	Intermarker distance	Marker	Change	FAM3	FAM3	FAM1	FAM1	FAM2	FAM2	61224	61224	61244	61244	61030	61030	61334	61334	61328	61328	61031	61031
107,600,949		rs3828085	**G > A,T**	**A**	**A**	**A**	**A**	**G**	**G**	**A**	**A**	**A**	**A**	**A**	**A**	**A**	**A**	**G**	**G**	**G**	**G**
107,605,964	5,015	rs9804074	**G > A**	**G**	**G**	**G**	**G**	**A**	**A**	**G**	**G**	**G**	**G**	**G**	**G**	**G**	**G**	**G**	**G**	**G**	**G**
107,639,225	33,261	rs6693140	**C > T**	**T**	**T**	**T**	**T**	**T**	**T**	**T**	**T**	**T**	**T**	**T**	**T**	**T**	**T**	**C**	**C**	**T**	**T**
107,642,995	3,770	rs4550085	**T > C**	**T**	**T**	**T**	**T**	**C**	**C**	**T**	**T**	**T**	**T**	**T**	**T**	**T**	**T**	**C**	**C**	**T**	**T**
107,647,631	4,636	rs12127467	**G > T**	**C**	**C**	**C**	**C**	**C**	**C**	**C**	**C**	**C**	**C**	**C**	**C**	**C**	**C**	**C**	**C**	**A**	**A**
107,665,660	18,029	rs4378232	**T > C**	**T**	**T**	**T**	**T**	**C**	**C**	**T**	**T**	**T**	**T**	**T**	**T**	**T**	**T**	**C**	**C**	**C**	**C**
107,701,229	35,569	rs12043814	**T > C**	**C**	**C**	**C**	**C**	**C**	**C**	**C**	**C**	**C**	**C**	**C**	**C**	**C**	**C**	**C**	**C**	**T**	**T**
107,702,788	1,559	rs2494066	**G > T,T**	**C**	**C**	**C**	**C**	**C**	**C**	**C**	**C**	**C**	**C**	**C**	**C**	**C**	**C**	**C**	**C**	**T**	**T**
107,704,426	1,638	rs2494070	**A > G**	**A**	**A**	**A**	**A**	**A**	**A**	**A**	**A**	**A**	**A**	**A**	**A**	**A**	**A**	**A**	**A**	**G**	**G**
107,708,701	4,275	rs12403629	**C > T**	**T**	**T**	**C**	**C**	**C**	**C**	**T**	**T**	**T**	**T**	**C**	**C**	**C**	**C**	**C**	**C**	**C**	**C**
107,726,008	17,307	rs4503376	**G > T,T**	**T**	**T**	**T**	**T**	**T**	**T**	**T**	**T**	**T**	**T**	**T**	**T**	**T**	**T**	**T**	**T**	**T**	**T**
107,726,179	171	rs17019810	**C > T**	**C**	**C**	**C**	**C**	**T**	**T**	**T**	**T**	**T**	**T**	**C**	**C**	**C**	**C**	**C**	**C**	**C**	**C**
107,739,098	12,919	rs7519428	**G > A**	**G**	**G**	**A**	**A**	**G**	**G**	**G**	**G**	**G**	**G**	**G**	**G**	**G**	**G**	**A**	**A**	**G**	**G**
107,762,256	23,158	rs4415601	**G > T**	**T**	**T**	**G**	**G**	**G**	**G**	**G**	**G**	**G**	**G**	**T**	**T**	**T**	**T**	**G**	**G**	**T**	**T**
107,772,515	10,259	rs4462178	**C > T**	**T**	**T**	**C**	**C**	**C**	**C**	**C**	**C**	**C**	**C**	**C**	**C**	**C**	**C**	**C**	**C**	**C**	**C**
107,782,599	10,084	rs10881483	**C > T**	**T**	**T**	**C**	**C**	**T**	**T**	**T**	**T**	**T**	**T**	**T**	**T**	**T**	**T**	**C**	**C**	**T**	**T**
107,788,674	6,075	rs11576720	**T > C**	**T**	**T**	**T**	**T**	**T**	**T**	**T**	**T**	**T**	**T**	**T**	**T**	**T**	**T**	**T**	**T**	**C**	**C**
107,811,048	22,374	rs17020104	**T > C**	**T**	**T**	**T**	**T**	**T**	**T**	**T**	**T**	**T**	**T**	**T**	**T**	**T**	**T**	**T**	**T**	**T**	**T**
107,813,378	2,330	rs17485,868	**A > G,T**	**G**	**G**	**A**	**A**	**G**	**G**	**G**	**G**	**G**	**G**	**A**	**A**	**A**	**A**	**G**	**G**	**G**	**G**
107,836,421	23,043	rs4460667	**G > A**	**G**	**G**	**G**	**G**	**A**	**A**	**A**	**A**	**A**	**A**	**G**	**G**	**G**	**G**	**A**	**A**	**A**	**A**
107,843,782	7,361	rs1380445	**T > G**	**T**	**T**	**T**	**T**	**G**	**G**	**G**	**G**	**G**	**G**	**T**	**T**	**-**	**-**	**G**	**G**	**G**	**G**
107,855,772	11,990	rs13373947	**A > G**	**A**	**A**	**A**	**A**	**G**	**G**	**G**	**G**	**G**	**G**	**A**	**A**	**A**	**A**	**A**	**A**	**A**	**A**
107,882,433	26,661	rs345292	**G > A**	**A**	**A**	**A**	**A**	**G**	**G**	**G**	**G**	**G**	**G**	**G**	**G**	**G**	**G**	**G**	**G**	**A**	**A**
107,888,320	5,887	rs11185201	**T > C**	**C**	**C**	**C**	**C**	**C**	**C**	**C**	**C**	**C**	**C**	**T**	**T**	**T**	**T**	**C**	**C**	**C**	**C**
107,899,020	10,700	rs17020294	**T > C**	**T**	**T**	**T**	**T**	**T**	**T**	**T**	**T**	**T**	**T**	**T**	**T**	**T**	**T**	**T**	**T**	**T**	**T**
107,905,580	6,560	rs10494083	**T > C**	**T**	**T**	**T**	**T**	**T**	**T**	**T**	**T**	**T**	**T**	**T**	**T**	**T**	**T**	**T**	**T**	**C**	**C**
107,924,764	19,184	rs345306	**T > G**	**G**	**G**	**T**	**T**	**T**	**T**	**T**	**T**	**T**	**T**	**G**	**G**	**G**	**G**	**T**	**T**	**T**	**T**
107,932,019	7,255	rs17020387	**C > T,G**	**C**	**C**	**C**	**C**	**C**	**C**	**C**	**C**	**C**	**C**	**C**	**C**	**C**	**C**	**C**	**C**	**C**	**C**
107,941,495	9,476	rs17020437	**T > C**	**T**	**T**	**C**	**C**	**C**	**C**	**C**	**C**	**C**	**C**	**T**	**T**	**T**	**T**	**C**	**C**	**T**	**T**
107,968,879	27,384	rs404634	**T > C**	**C**	**C**	**C**	**C**	**C**	**C**	**C**	**C**	**C**	**C**	**C**	**C**	**-**	**-**	**C**	**C**	**T**	**T**
107,988,189	19,310	rs12569255	**T > C**	**T**	**T**	**T**	**T**	**T**	**T**	**T**	**T**	**T**	**T**	**T**	**T**	**-**	**-**	**T**	**T**	**T**	**T**
107,995,780	7,591	rs12563016	**G > T**	**G**	**G**	**G**	**G**	**G**	**G**	**G**	**G**	**G**	**G**	**T**	**T**	**-**	**-**	**G**	**G**	**G**	**G**
108,011,539	15,759	rs1777467	**G > A**	**A**	**A**	**A**	**A**	**A**	**A**	**A**	**A**	**A**	**A**	**G**	**G**	**G**	**G**	**A**	**A**	**A**	**A**
108,029,484	17,945	rs4915093	**G > A**	**G**	**G**	**G**	**G**	**G**	**G**	**G**	**G**	**G**	**G**	**G**	**G**	**G**	**G**	**G**	**G**	**G**	**G**
108,071,266	41,782	rs1343192	**C > T**	**C**	**C**	**C**	**C**	**C**	**C**	**C**	**C**	**C**	**C**	**C**	**C**	**C**	**C**	**C**	**C**	**C**	**C**
108,073,978	2,712	rs10494087	**A > G**	**A**	**A**	**A**	**A**	**A**	**A**	**A**	**A**	**A**	**A**	**G**	**G**	**G**	**G**	**A**	**A**	**A**	**A**
108,092,662	18,684	rs12034547	**T > G**	**T**	**T**	**T**	**T**	**T**	**T**	**T**	**T**	**T**	**T**	**G**	**G**	**G**	**G**	**T**	**T**	**T**	**T**
108,098,283	5,621	rs2336126	**A > G**	**A**	**A**	**A**	**A**	**A**	**A**	**A**	**A**	**A**	**A**	**A**	**A**	**A**	**A**	**A**	**A**	**A**	**A**
108,111,706	13,423	rs501273	**G > T**	**A**	**A**	**A**	**A**	**A**	**A**	**A**	**A**	**A**	**A**	**A**	**A**	**A**	**A**	**A**	**A**	**A**	**A**
108,113,616	1,910	rs614499	**G > T**	**A**	**A**	**A**	**A**	**A**	**A**	**A**	**A**	**A**	**A**	**A**	**A**	**A**	**A**	**A**	**A**	**A**	**A**
108,119,014	5,398	rs1660419	**A > C**	**C**	**C**	**C**	**C**	**C**	**C**	**C**	**C**	**C**	**C**	**C**	**C**	**C**	**C**	**C**	**C**	**C**	**C**
108,120,673	1,659	rs1777450	**T > C**	**C**	**C**	**C**	**C**	**C**	**C**	**C**	**C**	**C**	**C**	**C**	**C**	**C**	**C**	**C**	**C**	**C**	**C**
108,137,612	16,939	rs17513833	**C > T**	**C**	**C**	**C**	**C**	**C**	**C**	**C**	**C**	**C**	**C**	**C**	**C**	**C**	**C**	**C**	**C**	**C**	**C**
108,168,646	31,034	rs829004	**T > A,C**	**C**	**C**	**C**	**C**	**C**	**C**	**C**	**C**	**C**	**C**	**C**	**C**	**C**	**C**	**T**	**T**	**T**	**T**
108,183,767	15,121	rs597999	**C > T**	**T**	**T**	**T**	**T**	**T**	**T**	**T**	**T**	**T**	**T**	**T**	**T**	**T**	**T**	**T**	**T**	**T**	**T**
108,198,443	14,676	rs594397	**A > G**	**A**	**A**	**A**	**A**	**A**	**A**	**A**	**A**	**A**	**A**	**A**	**A**	**A**	**A**	**A**	**A**	**A**	**A**
108,211,211	12,768	rs7538977	**T > C**	**T**	**T**	**T**	**T**	**T**	**T**	**T**	**T**	**T**	**T**	**T**	**T**	**T**	**T**	**C**	**C**	**C**	**C**
108,328,623	117,412	rs1417300	**T > C**	**T**	**T**	**T**	**T**	**T**	**T**	**T**	**T**	**T**	**T**	**T**	**T**	**T**	**T**	**C**	**C**	**C**	**C**
108,504,914	176,291	rs10776805	**G > A**	**G**	**G**	**G**	**G**	**G**	**G**	**G**	**G**	**G**	**G**	**G**	**G**	**G**	**G**	**G**	**G**	**G**	**G**
108,527,234	22,320	rs12036929	**C > T**	**C**	**C**	**C**	**C**	**C**	**C**	**C**	**C**	**C**	**C**	**C**	**C**	**C**	**C**	**C**	**C**	**C**	**C**
108,531,271	4,037	rs11101974	**T > C**	**T**	**T**	**T**	**T**	**T**	**T**	**T**	**T**	**T**	**T**	**T**	**T**	**T**	**T**	**T**	**T**	**T**	**T**
108,566,144	34,873	rs1353721	**G > A,T**	**A**	**A**	**A**	**A**	**A**	**A**	**A**	**A**	**A**	**A**	**A**	**A**	**A**	**A**	**A**	**A**	**A**	**A**
108,583,777	17,633	rs17024,373	**G > A**	**G**	**G**	**G**	**G**	**G**	**G**	**G**	**G**	**G**	**G**	**G**	**G**	**G**	**G**	**G**	**G**	**G**	**G**
108,588,401	4,624	rs970860	**A > C**	**C**	**C**	**C**	**C**	**C**	**C**	**C**	**C**	**C**	**C**	**C**	**C**	**C**	**C**	**C**	**C**	**C**	**C**
108,716,296	127,895	rs11102288	**G > T**	**C**	**C**	**C**	**C**	**C**	**C**	**C**	**C**	**C**	**C**	**C**	**C**	**C**	**C**	**C**	**C**	**C**	**C**
108,717,735	1,439	rs4970804	**G > A**	**A**	**A**	**A**	**A**	**A**	**A**	**A**	**A**	**A**	**A**	**A**	**A**	**-**	**-**	**A**	**A**	**A**	**A**
108,722,941	5,206	rs4970808	**C > T**	**T**	**T**	**T**	**T**	**T**	**T**	**T**	**T**	**T**	**T**	**T**	**T**	**T**	**T**	**T**	**T**	**T**	**T**
108,760,685	37,744	rs4970811	**A > G**	**G**	**G**	**G**	**G**	**G**	**G**	**G**	**G**	**G**	**G**	**G**	**G**	**G**	**G**	**A**	**A**	**A**	**A**
108,816,336	55,651	rs6696787	**T > C**	**C**	**C**	**C**	**C**	**C**	**C**	**C**	**C**	**C**	**C**	**C**	**C**	**C**	**C**	**T**	**T**	**T**	**T**
108,816,880	544	rs1277213	**A > G**	**G**	**G**	**G**	**G**	**G**	**G**	**G**	**G**	**G**	**G**	**G**	**G**	**G**	**G**	**G**	**G**	**G**	**G**
108,820,610	3,730	rs1333130	**T > A,C**	**T**	**T**	**T**	**T**	**T**	**T**	**T**	**T**	**T**	**T**	**T**	**T**	**T**	**T**	**C**	**C**	**C**	**C**
108,841,814	21,204	rs10857972	**C > T**	**C**	**C**	**C**	**C**	**C**	**C**	**C**	**C**	**C**	**C**	**C**	**C**	**C**	**C**	**C**	**C**	**C**	**C**
108,854,607	12,793	rs12405585	**G > T**	**C**	**C**	**C**	**C**	**C**	**C**	**C**	**C**	**C**	**C**	**C**	**C**	**C**	**C**	**C**	**C**	**C**	**C**
108,866,143	11,536	rs2131905	**C > T**	**C**	**C**	**C**	**C**	**C**	**C**	**C**	**C**	**C**	**C**	**C**	**C**	**C**	**C**	**C**	**C**	**C**	**C**
108,943,574	77,431	rs338466	**A > G,T**	**A**	**A**	**A**	**A**	**A**	**A**	**A**	**A**	**A**	**A**	**A**	**A**	**A**	**A**	**A**	**A**	**A**	**A**
**108,950,376**	6,802	CLCC1-MUTATION	**G > T**	**T**	**T**	**T**	**T**	**T**	**T**	**T**	**T**	**T**	**T**	**T**	**T**	**T**	**T**	**T**	**T**	**T**	**T**
108,963,067	12,691	rs550743	**T > A,C**	**C**	**C**	**C**	**C**	**C**	**C**	**C**	**C**	**C**	**C**	**C**	**C**	**C**	**C**	**C**	**C**	**C**	**C**
108,970,652	7,585	rs11803800	**A > G**	**A**	**A**	**A**	**A**	**A**	**A**	**A**	**A**	**A**	**A**	**A**	**A**	**A**	**A**	**A**	**A**	**A**	**A**
108,972,406	1,754	rs12032662	**A > C,G**	**C**	**C**	**C**	**C**	**C**	**C**	**C**	**C**	**C**	**C**	**C**	**C**	**C**	**C**	**C**	**C**	**C**	**C**
108,973,113	707	rs570812	**A > G**	**A**	**A**	**A**	**A**	**A**	**A**	**A**	**A**	**A**	**A**	**A**	**A**	**A**	**A**	**A**	**A**	**A**	**A**
109,142,651	169,538	rs587727	**A > G,T**	**G**	**G**	**A**	**A**	**G**	**G**	**G**	**G**	**G**	**G**	**G**	**G**	**G**	**G**	**G**	**G**	**G**	**G**
109,154,346	11,695	rs595635	**A > G**	**A**	**A**	**A**	**A**	**A**	**A**	**A**	**A**	**A**	**A**	**A**	**A**	**A**	**A**	**A**	**A**	**A**	**A**
109,171,781	17,435	rs17014,495	**G > T**	**A**	**A**	**A**	**A**	**A**	**A**	**C**	**C**	**C**	**C**	**C**	**C**	**C**	**C**	**C**	**C**	**C**	**C**
109,196,449	24,668	rs2478762	**T > A,C**	**C**	**C**	**C**	**C**	**C**	**C**	**C**	**C**	**C**	**C**	**C**	**C**	**C**	**C**	**C**	**C**	**C**	**C**
109,197,926	1,477	rs2296696	**G > T**	**T**	**T**	**T**	**T**	**T**	**T**	**T**	**T**	**T**	**T**	**T**	**T**	**T**	**T**	**T**	**T**	**T**	**T**
109,206,449	8,523	rs3197233	**T > C**	**C**	**C**	**C**	**C**	**C**	**C**	**C**	**C**	**C**	**C**	**C**	**C**	**C**	**C**	**C**	**C**	**C**	**C**
109,209,582	3,133	rs604500	**T > C**	**C**	**C**	**T**	**T**	**C**	**C**	**C**	**C**	**C**	**C**	**C**	**C**	**C**	**C**	**C**	**C**	**C**	**C**
109,229,079	19,497	rs669697	**A > C**	**C**	**C**	**A**	**A**	**C**	**C**	**C**	**C**	**C**	**C**	**C**	**C**	**C**	**C**	**C**	**C**	**C**	**C**
109,240,933	11,854	rs4246519	**A > G**	**A**	**A**	**A**	**A**	**A**	**A**	**A**	**A**	**A**	**A**	**A**	**A**	**A**	**A**	**A**	**A**	**A**	**A**
109,246,091	5,158	rs17035,443	**G > A**	**G**	**G**	**A**	**A**	**G**	**G**	**G**	**G**	**G**	**G**	**G**	**G**	**G**	**G**	**G**	**G**	**G**	**G**
109,262,024	15,933	rs4970833	**G > A**	**G**	**G**	**G**	**G**	**G**	**G**	**G**	**G**	**G**	**G**	**G**	**G**	**G**	**G**	**G**	**G**	**G**	**G**
109,289,661	27,637	rs655246	**A > C,G**	**G**	**G**	**A**	**A**	**G**	**G**	**A**	**A**	**A**	**A**	**A**	**A**	**A**	**A**	**A**	**A**	**A**	**A**
109,338,099	48,438	rs11102972	**T > C**	**T**	**T**	**T**	**T**	**T**	**T**	**T**	**T**	**T**	**T**	**T**	**T**	**T**	**T**	**T**	**T**	**T**	**T**
109,369,429	31,330	rs17646665	**A > G**	**A**	**A**	**A**	**A**	**A**	**A**	**A**	**A**	**A**	**A**	**A**	**A**	**A**	**A**	**A**	**A**	**A**	**A**
109,381,055	11,626	rs12037569	**G > T**	**G**	**G**	**G**	**G**	**G**	**G**	**T**	**T**	**T**	**T**	**T**	**T**	**T**	**T**	**T**	**T**	**T**	**T**
109,473,110	92,055	rs10494040	**C > T**	**T**	**T**	**T**	**T**	**T**	**T**	**T**	**T**	**T**	**T**	**T**	**T**	**T**	**T**	**T**	**T**	**T**	**T**
109,505,814	32,704	rs3738772	**C > T,A**	**C**	**C**	**C**	**C**	**C**	**C**	**C**	**C**	**C**	**C**	**C**	**C**	**C**	**C**	**C**	**C**	**C**	**C**
109,513,504	7,690	rs6677291	**C > T**	**C**	**C**	**C**	**C**	**C**	**C**	**T**	**T**	**T**	**T**	**T**	**T**	**T**	**T**	**T**	**T**	**T**	**T**
109,519,003	5,499	rs534135	**T > C**	**C**	**C**	**T**	**T**	**C**	**C**	**C**	**C**	**C**	**C**	**C**	**C**	**C**	**C**	**C**	**C**	**C**	**C**
109,778,808	259,805!	rs12239350	**T > C**	**-**	**-**	**-**	**-**	**-**	**-**	**C**	**C**	**T**	**T**	**T**	**T**	**T**	**T**	**C**	**C**	**C**	**C**
109,883,546	104,738	rs875903	**G > A**	**-**	**-**	**-**	**-**	**-**	**-**	**G**	**G**	**A**	**A**	**G**	**G**	**G**	**G**	**G**	**G**	**G**	**G**
110,000,402	116,856	rs720917	**T > C**	**-**	**-**	**-**	**-**	**-**	**-**	**T**	**T**	**T**	**T**	**T**	**T**	**T**	**T**	**T**	**T**	**T**	**T**
110,104,236	103,834	rs11576956	**A > G**	**-**	**-**	**-**	**-**	**-**	**-**	**G**	**G**	**A**	**A**	**G**	**G**	**G**	**G**	**A**	**A**	**G**	**G**
110,234,421	130,185	rs11102065	**C > T**	**-**	**-**	**-**	**-**	**-**	**-**	**C**	**C**	**T**	**T**	**C**	**C**	**C**	**C**	**T**	**T**	**C**	**C**
110,278,256	43,835	rs752653894	**T > C,G**	**-**	**-**	**-**	**-**	**-**	**-**	**T**	**T**	**T**	**T**	**T**	**T**	**T**	**T**	**T**	**T**	**T**	**T**
110,330,874	52,618	rs61787370	**T > C**	**-**	**-**	**-**	**-**	**-**	**-**	**T**	**T**	**C**	**C**	**T**	**T**	**T**	**T**	**C**	**C**	**T**	**T**
110,472,417	141,543	rs11102121	**C > T**	**-**	**-**	**-**	**-**	**-**	**-**	**C**	**C**	**C**	**C**	**C**	**C**	**C**	**C**	**C**	**C**	**T**	**T**
110,628,756	156,339	rs2640491	**C > T**	**-**	**-**	**-**	**-**	**-**	**-**	**C**	**C**	**T**	**T**	**T**	**T**	**-**	**-**	**T**	**T**	**C**	**C**
111,136,919	508,163	rs1030926216	**T > G**	**-**	**-**	**-**	**-**	**-**	**-**	**T**	**T**	**G**	**G**	**T**	**T**	**-**	**-**	**T**	**T**	**G**	**G**

### Haplotype Analysis and Age Estimation

SNP haplotypes in individuals carrying the c.75C > A mutation were delineated by examination as most individuals were homozygous for the region immediately surrounding the mutation. The haplotype was extended until only two families maintained the original founder haplotype. The ancestral haplotype was determined by identifying the common haplotype shared by all patients and extending the founder haplotype as the adjacent SNP genotypes shared by the largest number of samples. In 3 cases two alternate haplotypes diverging at the same SNP were shared by two families, and those pairs were considered to share a common ancestor at the divergence point. Haplotypes were sorted into those requiring one or two recombination events in their descent from the ancestral haplotype by examination, minimizing the number of recombination events required (Jiao et al., 2017).

The mutation age in generations was estimated using three independent but related approaches. The first estimation was performed as described in Equation (Hartong et al., 2006) of Risch et al. (Risch et al., 1995; Colombo, 2000): *g* = log(*δ*)/log (1- *δ*), where *δ* is the linkage disequilibrium constant, *δ*=(P_D_ − P_N_)/(1− P_N_), with P_D_ being the frequency of the allele on chromosomes of mutation carriers and P_N_ being the frequency of the allele in the control population. Genotypes of unaffected individuals were taken from the 1,000 Genomes database (96 samples in the South Asian population) (Genomes Project et al., 2015). Haplotypes were estimated using the EM (expectation maximization) and CHM (composite haplotype) methods as implemented in the Golden Helix SVS (Golden Helix, Bozeman, MT, United States). The map distances were inferred on the basis of the physical distances as given in GRCh38/hg38 from the UCSC Genome Browser assuming 1 Mb corresponds to 1 cM. Alleles at SNPs showing no recombination in the cases were collapsed into a single haplotype block and analyzed as a single marker in the two markers approach as described in Equation (Bird, 1995) of Risch et al.

In the second approach, marker specific values of g were estimated using the iterative method described in Equation (Hartong et al., 2006) of Goldstein et al. (Goldstein et al., 1999): K = cR + μM+(1-c-μ)I, where *c* and *μ* are the recombination and mutation rates, respectively, R is a 2 × 2 matrix with R11 = R12 = a, and R21 = R22 = 1 − a, where a is the frequency of the ancestral allele in the control population, M is a 2 × 2 matrix of mutation probabilities with M11 = 0, M12 = 1/3, M21 = 1, and M22 = 2/3, modified from Goldstein et al. to account for the frequency with which a mutation in a SNP might remove an ancestral allele (all possible bases) or move to an ancestral allele (1 of three possible bases), and I is the 2 × 2 identity matrix. The original frequency vector is (1, 0) as it occurs on the founder haplotype, and this association is reduced by multiplying by K at each generation (*g*) until the current frequency of the ancestral allele is reached. Once more, alleles at SNPs showing no recombination in the cases were collapsed into a single haplotype block and analyzed as a single marker.

In the third approach, the marginal posterior probability distribution of the age (Slatkin and Rannala, 2000) of the c.75C > A mutation was estimated using the DMLE+2.3 software developed by Reeve and Rannala (Reeve and Rannala, 2002). This program estimates the age in generations by comparing the observed haplotypes in chromosomes from affected and unaffected sample sets considering the map distances, the population growth rate (^gen^ *r*), and the proportion of the mutation bearing chromosomes sampled, but has a strong dependence on assumptions regarding the population history of Pakistan. Somewhat arbitrarily, population growth was modeled using growth rates of 0.5, 1.0, 1.5 and 2.0%, as the population history of Pakistan and especially the Indus valley, from which most of our families are drawn, is complicated by a decline around 1,800 BC, the influx of new populations from around 2,000–1,500 BC, and invasion by a number of foreign powers including the army of Alexander the Great, the Arab and Muslim conquests and the British Indian Empire, each of which impacted the population number and composition.

In the final approach the estimate used the Gamma method as described by Gandolfo et al. (Gandolfo et al., 2014) as implemented in the WEHI Bioinformatics website: https://shiny.wehi.edu.au/rafehi.h/mutation-dating/. It is based on the length of conserved ancestral segments surrounding the mutation, and requires few additional assumptions, being specifically applicable for use with dense SNP haplotypes and small sample sizes.

## Results

### Families With the c.75C > A Mutation

**FIGURE 1:**
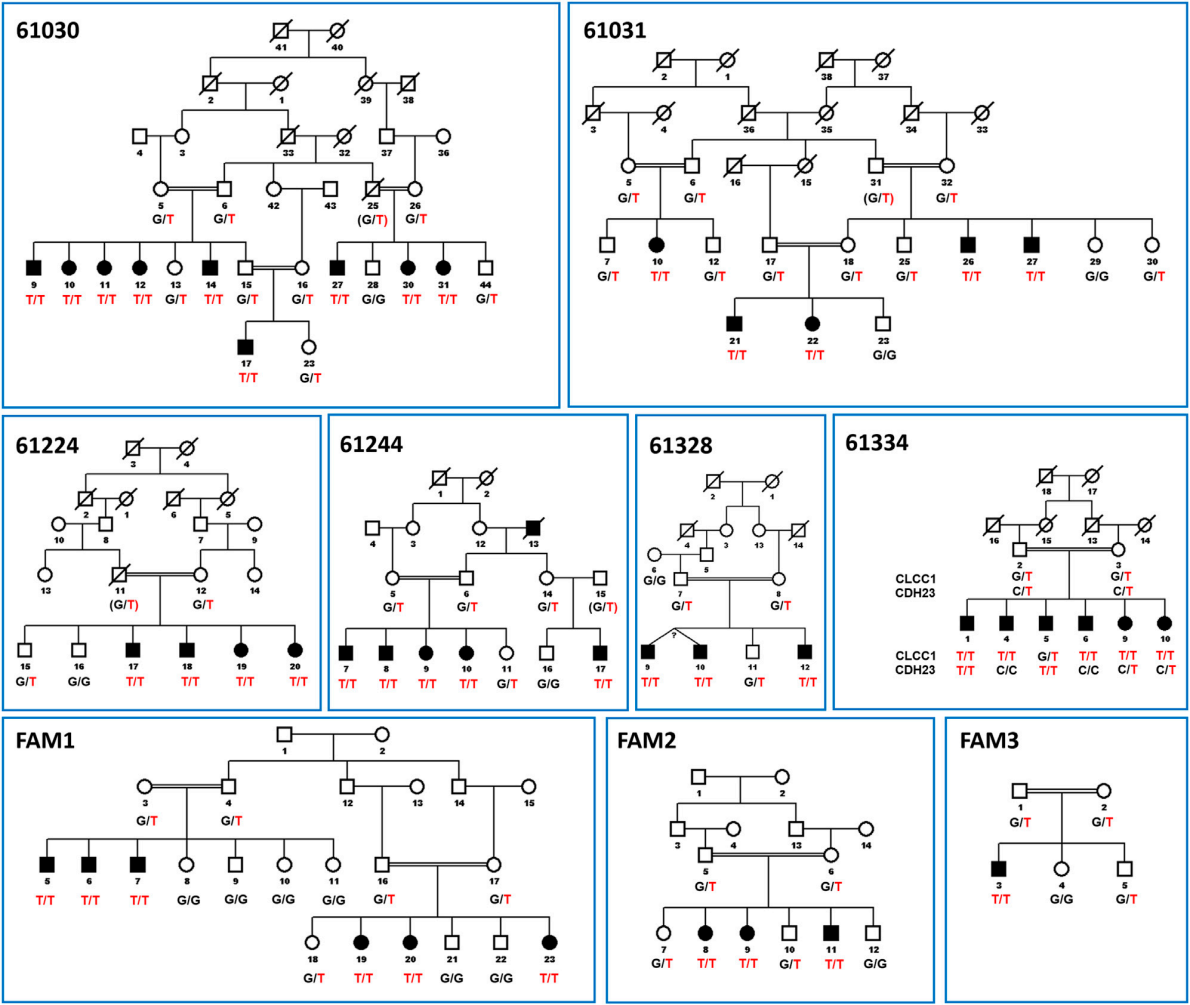
Pedigree figures of CLCC1 mutation in the nine consanguineous families with genotypes of available individuals. The squares and the circles represent males and females, respectively. The black-filled symbols indicate patients with retinitis pigmentosa, and a symbol with a diagonal line indicates a deceased family member. The candidate variants are listed under each pedigree, and the genotypes of the individuals for the variants are marked below each symbol. The c.75C > A mutation is shown in the figure as NC_000001.11:g. 108950376G > T for consistency with the genomic description of the SNP in databases and in Figure 2. “T/T” indicates homozygous variant, “G/T” or “C/T” indicate heterozygous variants, and “G/G” or “C/C” indicates homozygous reference alleles for *CLCC1* and *CDH23*, respectively. CDH23 alleles are shown for Family 61334 as individual five is affected on the basis of inheriting two variant alleles for CDH23 and is only heterozygous for the CLCC1 variant allele, while individual one in that family is homozygous for both variants. Linkage results and SNP haplotypes are shown in Li et al. (Li et al., 2018).

The present study includes nine RP families carrying the c.75C > A mutation. The majority of families, including FAM1, FAM2, 61224, 61244, 61030, 61328 and 61031 from Pakistan as well as a British-Bangladeshi family (FAM3), have been described previously (Li et al., 2018). One new family from Pakistan with the CLCC1 mutation (61334) has been identified more recently and met the same criteria for diagnosis of arRP. The SNP haplotypes of the affected families are shown in Table 1 and the family structures are shown in Figure 1.

Whole genome sequencing and SNP mapping of these families initially identified a 1.5 Mb region of homozygosity on chromosome 1p13.3 common to affected family members of all nine families (flanked by markers rs3828085 and rs1030926216), suggesting a single ancestral founder mutation as the cause of the condition. Further SNP mapping of all individuals in all nine families narrowed the autozygous region common in all affected family members to a small 158 kb interval flanked by rs10857972-rs570812 (chr1: 108, 814, 814–108,973,113, hg38). All affected members of all the families shared the identical SNP haplotype (Table 1), which is estimated to have a frequency in the Pakistani population (Lahore) of 0.01 by the EM algorithm as incorporated into the Golden Helix SVS program (Bozeman MT), strongly suggesting that arRP in all nine families arose due to autozygosity for the same ancestral mutation (p = 2x10^−11^). This includes the British-Bangladeshi family (FAM3), which is also likely to be of Pakistani (Punjab) origin.

### Haplotype Analysis

**FIGURE 2:**
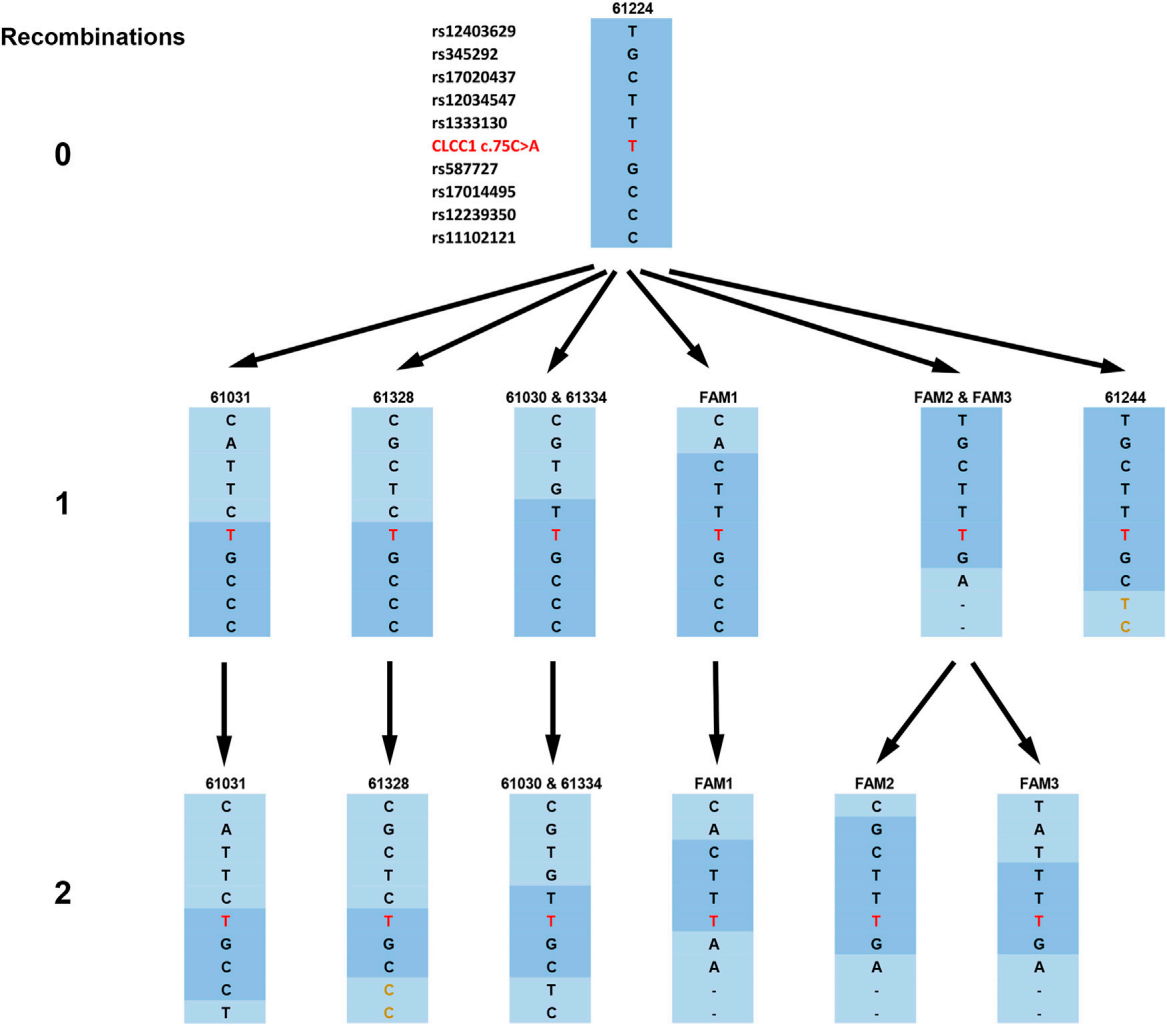
Haplotypes of the families and their derivation from the original risk haplotype, as shown by the compressed haplotype blocks labeled by the first SNP from the c.75C > A mutation (here shown in the figure as NC_000001.11:g. 108950376G > T for consistency with other SNPs). The original risk haplotype is shown in dark blue, and recombined haplotype blocks are shown in light blue. The mutation is shown in red and haplotypes in families 61328 and 61244 with the identical initial allele but part of a haplotype differing from those families 61224 (the founder haplotype) and 61030/61334 respectively are shown in gold.

The SNP haplotypes from the Pakistan arRP patients harboring the c.75C > A mutation included in this study were extended until the common haplotype was lost (that is, only two families showed the identical conserved haplotype) and aligned as shown in Table 1. The haplotype shaded in blue in homozygotes was the most common in affected members of our families and was assumed to be the ancestral haplotype. Divergent regions of the patient haplotypes are unshaded, except for three pairs of two families each (FAM2 and FAM3; 61030 and 61334; 61031 and 61328), in which the haplotype diverge at the same SNP, and the divergent haplotypes are identical for each pair beyond the divergent SNP. These families are assumed to represent offspring of a single divergent ancestor and are colored various shades of green. Two arRP associated mutations are segregating in family 61334 (Figure 2). The CLCC1 c.75G > T mutation is homozygous in individuals 1, 4, 6 and 10, and the CDH23 c.1595C > T mutation is homozygous in individuals 1 and 5, so that individual 1 has arRP on the basis of mutations in both genes.

The arRP-associated haplotypes from the nine affected individuals are shown in Figure 2, ordered into three levels based on the number of recombination events required to derive each haplotype from the founder haplotype, which is shown at the top (level 0). Haplotype blocks are designated by the first SNP from the c.75C > A mutation in the block. A single recombination event can generate the haplotypes shown in level 1, four different recombination events (one in families 61030 and 61334, a second in families 61031 and 61328 and a third in FAM1) above and two (one in FAM2 and FAM3 and one in 61244) below the mutation. From five of these haplotypes the remainder, seen on level 2, can be generated by a second recombination on the opposite side of the mutation from the first. Thus, all haplotypes bearing the NC_000001.11:g. 108950376G > T mutation can be generated from the founder haplotype by a maximum of two recombination events, with family 61224 maintaining the founder haplotype and 61244 showing only a single recombination.

### Age Estimation of the c. 75C > A CLCC1 Mutation

**FIGURE 3:**
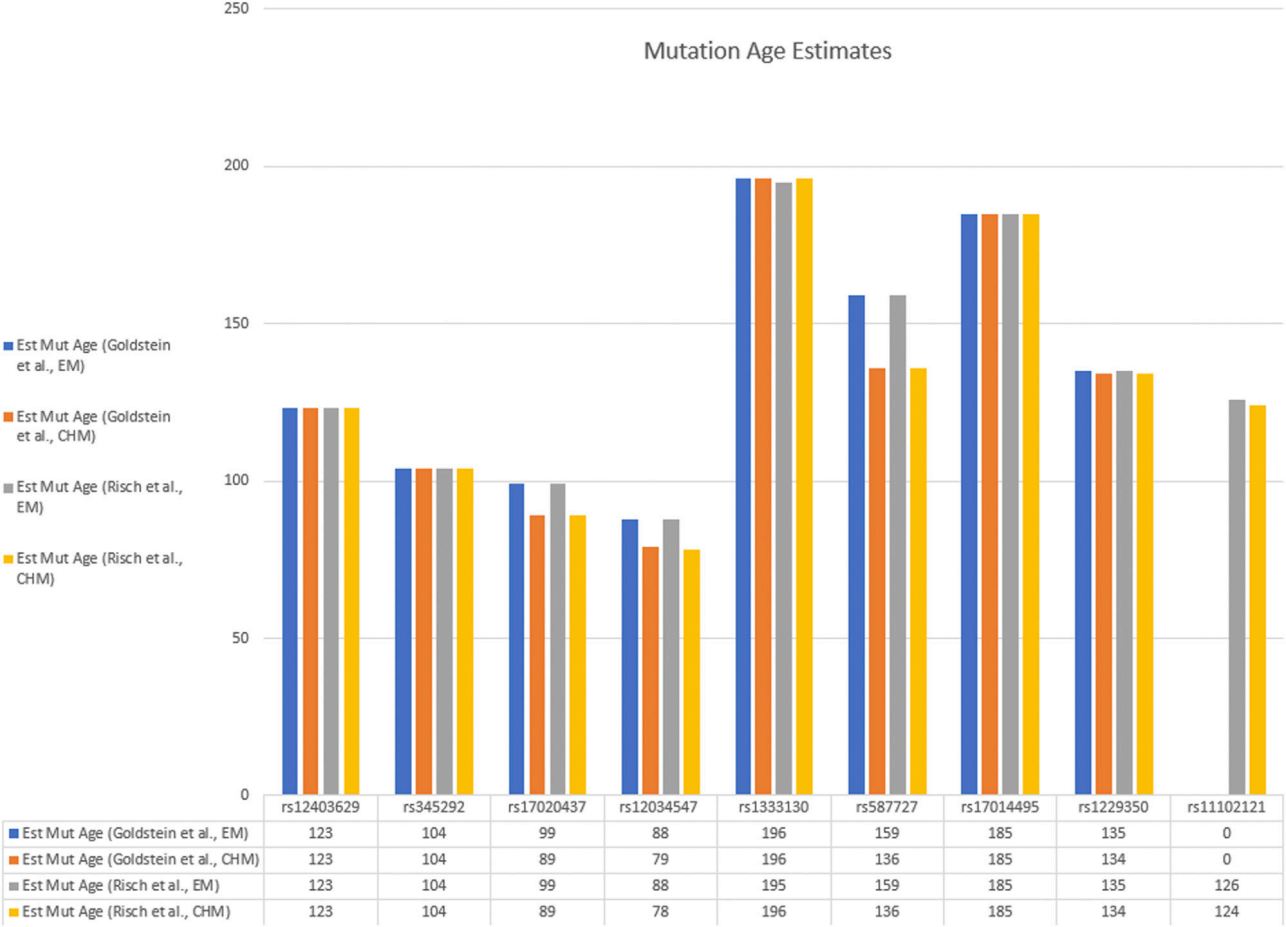
Bar graph of estimated mutation ages from various haplotype blocks by the Goldstein and Risch methods. Data are taken from Table 2.

**FIGURE 4:**
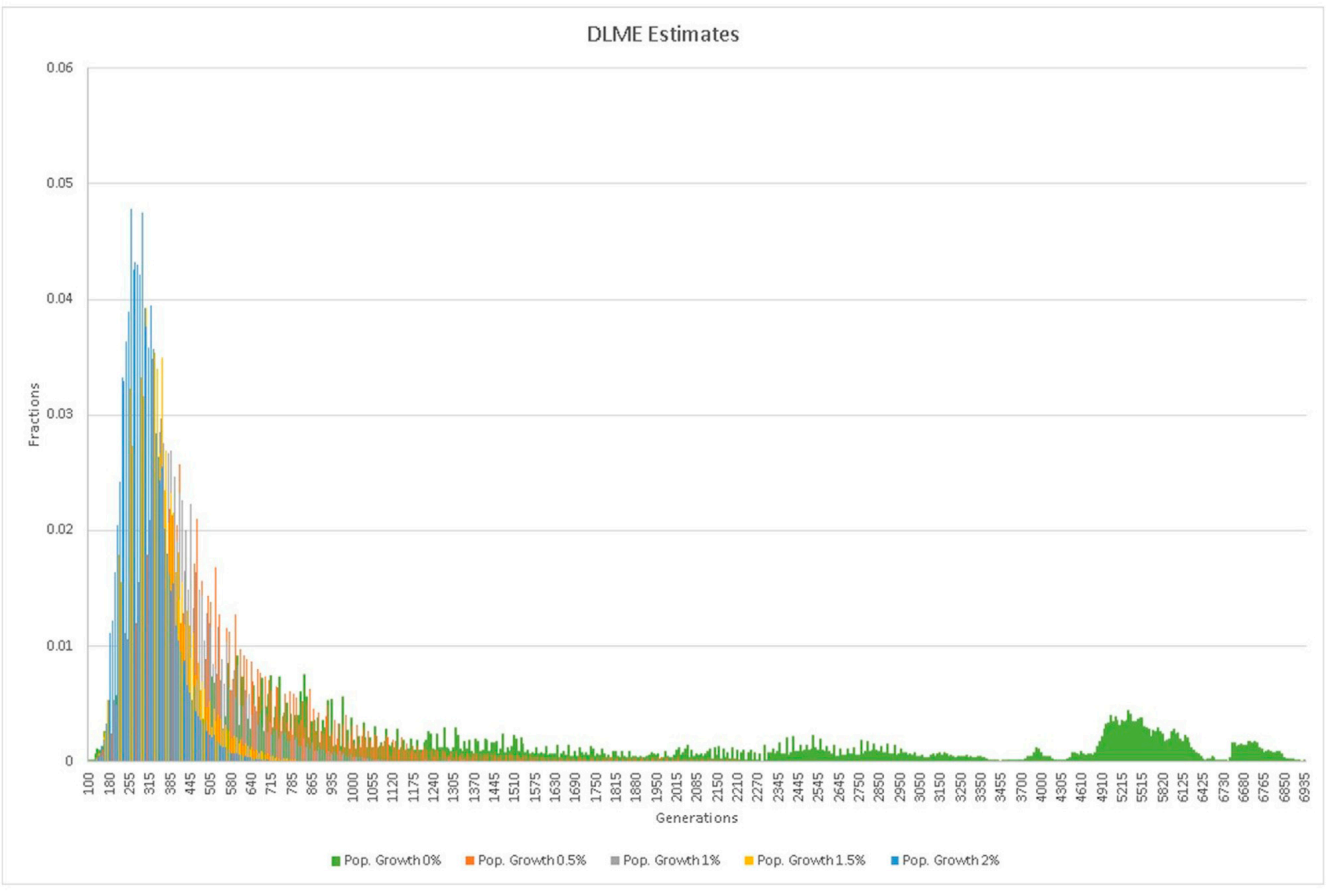
DMLE estimates of the mutation age assuming various rates of population growth.

The age of the c.75C > A mutation was estimated in the Pakistan population using the approaches described by Risch et al. (Risch et al., 1995; Colombo, 2000), and Goldstein et al. (Goldstein et al., 1999), as well as Bayesian disequilibrium estimates of the marginal posterior density as instituted in the DMLE+2.3 program (Reeve and Rannala, 2002). A summary of these results with the age given in generations is shown in Tables 2, 3 and Figures 3, 4. Using the multiple marker approach method described by Risch et al., with nonrecombinant markers combined into haplotype blocks provides estimates ranging from 79 (1975 years) to 196 (4,900 years) generations. Using the method described by Goldstein et al., similar ranges were obtained for the shared haplotypes: 79 to 196 generations (1975–4,900 years). It can be seen that similar estimates were obtained with the Risch and Goldstein approaches, although there is some variability depending on how the haplotype frequencies were estimated, and significant variability depending on which haplotype block was used to estimate the age.

**TABLE 2:** Mutation age estimates in generations of the c.75C > A mutation in the South Asian population by the methods described by Goldstein et al., Risch et al., by Bayesian estimation, and Gamma estimation. Fn(EM): frequency of the observed haplotype in the normal population as estimated by the expectation-maximization method, Fn(CHM): frequency of the observed haplotype in the normal population as estimated by the composite haplotype method, Theta: estimated recombination frequency of the haplotype block to the mutation, Pr (EM): probability of the haplotype in the affected family haplotypes as estimated by the EM method, Pr(CHM): probability of the haplotype in the affected family haplotypes as estimated by the CHM method, Gamma Ind: estimation from segment lengths assuming an ‘independent’ genealogy, Gamma Cor: Assuming a correlated genealogy. The c.75C > A mutation is alternately referred to as NC_000001.11:g.108950376G > T for consistency with other SNPs.

**Proximal SNP**	**rs12403629**	**rs345292**	**rs17020437**	**rs12034547**	**rs1333130**	**g.108950376 G **> T	**rs587727**	**rs17014,495**	**rs1229350**	**rs11102121**
Samples with Shared Haplotype	4	6	8	10	14	18	16	12	6	2
Fn (EM)	0.01042	0.01042	0.12489	0.16613	0.01042		0.57753	0.01042	0.01238	0.22394
Fn (CHM)	0.01042	0.01042	0.06510	0.09782	0.01042		0.51823	0.01042	0.00977	0.22656
Theta	0.01242	0.01068	0.01009	0.00858	0.00130	0	0.00192	0.00221	0.00828	0.01522
SNPs in Collapsed Haplotype	10	13	6	8	24	9	2	17	4	2
Pr (EM)	0.06781	0.00134	0.26401	0.16613	0.00027		0.19910	0.00010	0.08626	0.51046
Pr (CHM)	0.06781	0.00023	0.27172	0.17319	0.00027		0.31771	0.00010	0.14123	0.50521
Est Mut Age (Goldstein et al., EM)	123 (108–143)	104 (90–120)	99 (84–116)	88 (73–106)	196 (152–243)		159 (104–219)	185 (151–220)	135 (109–167)	N/A
Est Mut Age (Goldstein et al., CHM)	123 (108–143)	104 (90–120)	89 (76–104)	79 (66–93)	196 (152–243)		136 (90–187)	185 (151–220)	134 (109–167)	N/A
Est Mut Age (Risch et al., EM)	123 (108–143)	104 (91–120)	99 (84–118)	88 (73–106)	196 (152–243)		159 (104–219)	185 (151–222)	135 (109–168)	126 (108–149)
Est Mut Age (Risch et al., CHM)	123 (108–143)	104 (91–120)	89 (76–104)	79 (66–93)	196 (152–243)		136 (90–187)	185 (151–222)	134 (109–167)	124 (107–147)
Est Mut Age DMLE (1% pop growth)						309 (116–326)				
Est Mut Age Gamma Ind						104.3 (75.1–145.0)				
Est Mut Age Gamma Cor						99.4 (58.2–171.0)				

Fn: estimated frequency of the risk haplotype in the general population, EM: expectation-maximization, CHM: composite haplotype method.

Pr: probability of a mutation reverting a nonrisk haplotype to the risk haplotype, Est Mut Age: estimated mutation age by methods described in Goldstein et al.

Or Risch et al., Gamma Ind: estimation from segment lengths assuming an independent’ genealogy, Gamma Cor: Assuming a correlated genealogy.

**TABLE 3:** Summary of DMLE mutation age estimates for different population growth rates and their confidence intervals.

Population Growth Rate (%)	Mean Generations	Median Generations	95% Confidence Interval (Low-High)
0	2,160	1776	211–5,924
0.5	723	688	168–964
1	504	494	146–562
1.5	349	346	124–387
2	309	309	116–326

Analysis of the mutation age (Tables 2,3; Figure 4) using DMLE+2.3 showed that variations in the probability of an independent identical mutation does not have a major influence on the age estimate, whereas variations in the assumed growth rate of the Pakistani population is crucial. When the assumed population growth rate was 0%, the mean age estimate was 2,160 generations (95% CI: 211–5,924). When the population growth rate was increased to 2%, the mean estimated mutation age fell to 309 generations (95% CI: 116–326). The median estimated mutation age was somewhat lower than the mean, reflecting the effect of the skewed distributions, greater at lower population growth rates. Estimation of the mutation age was also carried out using the Gamma method as proposed by Gandolfo et al. (Gandolfo et al., 2014), based on ancestral segment lengths estimated from high density SNP data (Table 2). While the estimates (104 generations for independent ancestry since the founder and 99 generations if some sampled individuals have common ancestors since the founder) were somewhat lower than those obtained with DMLE, they still have overlapping confidence limits and are similar to some of the lower estimates from the Risch and Goldstein approaches.

## Discussion

In the current study, we have investigated nine unrelated Pakistani derived arRP patients (and families) carrying an identical c.75C > A CLCC1 mutation. A common shared haplotype of 99 microsatellite markers covering a region of approximately 3.5 Mb surrounding the c.75C > A CLCC1 mutation on chromosome 1p13.3 clearly indicates that these families originate from a common ancestor, with a p < 2x10^−11^ for the original eight families reported (Li et al., 2018) and p < 6x10^−13^ with the additional family reported here. Depending on different assumptions in the model related to assured phases in families, mutation frequencies at the different SNPs, recombination frequencies between SNP markers, and the likelihood of an independent identical CLCC1 mutation occurring in these families, we estimate that the mutation may have originated from 79 to 196 generations, or approximately 1975–4,900 years ago, if an average generation is assumed to be 25 years (Risch et al., 1995). A Bayesian approach yielded somewhat older estimates (309–2,160 generations), although the wide 95% confidence limits resulted in overlap of the ranges of the predictions for most analyses.

CLCC1 is transmembrane protein functioning as an anion and particularly chloride channel in the ER and possibly the Golgi apparatus and nucleus in some cell types (Nagasawa et al., 2001; Jia et al., 2015; Li et al., 2018). Notably, downregulation of this gene sensitizes cultured cells to chemically induced ER stress. Furthermore, loss of CLCC1 function *in vivo* results in upregulated expression of UPR target genes and the accumulation of ubiquitin-positive inclusions in neurons before their degeneration, suggesting that disruption of chloride/anion concentrations in the ER leads to loss of ER homeostasis and eventual neuron death (Jia et al., 2015). CLCC1 appears to interact with the mitochondrial microprotein PIGBOS to prevent induction of the UPR and cell death (Chu et al., 2019). Similarly, knockdown of CLCC1 by siRNA results in apoptotic cell death in cultured ARPE19 cells (Li et al., 2018). Li et al. (Li et al., 2018) previously identified the homozygous missense alteration (c.75C > A, p. D25E) in CLCC1 associated with autosomal recessive retinitis pigmentosa (arRP) in eight consanguineous families of Pakistani descent that were suggested to originate from a common founder on the basis of sharing a common SNP haplotype in the CLCC1 gene region.

The c.75C > A CLCC1 mutation is a moderately common cause of arRP in the Pakistan population and so far has been identified in nine different families from or originating in the Punjab region of Pakistan and thus being the cause of arRP in roughly 6% of families (Li et al., 2017). While the relatively short length of the haplotype shared by the affected families suggested that the original mutation might be old, incomplete haplotypes and lack of recombinant SNP genotypes at the ends of the haplotype in some families prevented a formal estimation of the mutation age. Completion and extension of the SNP haplotypes in all family members allowed estimation of the population age and history of the c.75C > A mutation. The mutation age was estimated using four approaches, all based on the recombination frequencies between the mutation and markers in affected individuals, marker mutation rates, allele frequencies in the general population and affected family members, and the genetic distance of markers from the mutation. These included an analytical approach as described by Risch et al. (Risch et al., 1995), an iterative approach as described by Goldstein et al. (Goldstein et al., 1999), a Gamma method based on SNP haplotypes (Gandolfo et al., 2014) and a posterior Bayesian distribution approach as implemented in the program DMLE+2.3 (Reeve and Rannala, 2002).

The estimates made using the first two approaches agreed closely, usually to within a generation for each marker. There was some variation generated by the estimated haplotype frequencies (using the CHM or EM method), although it was relatively small for most markers, usually within 10–15% of the estimated mutation age. The mutation rates, and especially the back-mutation rates, are much smaller for SNP markers than for microsatellites and have only a minimal effect, especially as there is no large variation influenced by the number of repeat units, as occurs with microsatellites (Chakraborty et al., 1997; Eckert and Hile, 2009). As is apparent from Tables 2, 3, the major source of variation lies in uncertainty regarding the recombination frequencies between each haplotype block and the c.75C > A mutation. This probably relates to the conversion value of 1 Mb ≈ 1 cM, which is only approximate, and can vary significantly in different regions of the chromosomes as well as between males and females (Broman et al., 1998).

Estimation of the mutation age using a posterior Bayesian distribution, while using all the information in the haplotypes, was excruciatingly dependent on the population growth estimates. These are difficult to ascertain, especially since the population growth of the Punjab region has not been constant over the estimated age of the mutation. Additionally, populations derived from a region including southwestern Iran flourished in the Punjab region until approximately 1800 BC, at which time the population constricted, coinciding with the influx of new population groups from the central Asian steppes (McElreavey and Quintana-Murci, 2005; Narasimhan et al., 2019). In addition, the region experienced subsequent invasions including those of Alexander the Great, the Umayyad and later Arab and Muslim conquests.

Each of these would have impacted the Indus valley population growth and composition in ways that are difficult to predict. For this reason, DMLE modeling was carried out under a series of assumed population growth rates ranging from 0 to 2%. The estimated mutation age was maximal at 2,160 generations under the 0% growth model, decreasing to 309 as the assumed growth rate was set at 2%, a reasonable estimate of the population growth of Pakistan as a whole since 1900, although growth in some recent years has been higher, especially between 1965 and 2005 (World Population Prospect, 2019). Finally, the Gamma method is particularly appealing for our families since it is intended for use with small sample numbers and, being based solely on the length of the conserved ancestral segment as estimated by a dense SNP haplotype, requires few assumptions regarding population structure and growth rate. In our families, as indicated in Figure 2, the analysis based on a correlated ancestry is probably the correct one to use, but the independent analysis is included for comparison, and the two estimates are actually quite close.

There is the possibility that the high rate of consanguinity in the Pakistan population might affect the mutation age estimates by all approaches. While recombination events would be undetectable in individuals homozygous for the risk allele (and thus affected) decreasing the apparent recombination rate, these would be fairly infrequent as affected individuals comprise only one fourth of the offspring of two heterozygous individuals, and as can be seen from the pedigree structure most matings in our families are not between two homozygotes, even though the pedigrees are selected for having a high number of affected individuals.

In conclusion, our results provide evidence that the c.75C > A mutation in the CLCC1 gene is significantly associated with arRP in Pakistan population. Moreover, haplotype analysis using high-density SNP data, as well as variant age estimates, strongly support a founder origin and linear pattern of descent for this mutation instead of multiple independent occurrences. The identification of founder mutation, such as the one here reported, may contribute for the development of more cost-efficient screening.

## Data Availability

The original contributions presented in the study are included in the article/Supplementary Material, further inquiries can be directed to the corresponding author.

## References

[B1] Bird A. C. (1995). Retinal Photoreceptor Dystrophies LI. Edward Jackson Memorial Lecture. Am. J. Ophthalmol. 119 (5), 543–562. 10.1016/s0002-9394(14)70212-0 7733180

[B2] Bittles A. (2001). Consanguinity and its Relevance to Clinical Genetics. Clin. Genet. 60 (2), 89–98. 10.1034/j.1399-0004.2001.600201.x 11553039

[B3] Broman K. W. Murray J. C. Sheffield V. C. White R. L. Weber J. L. (1998). Comprehensive Human Genetic Maps: Individual and Sex-specific Variation in Recombination. Am. J. Hum. Genet. 63 (3), 861–869. 10.1086/302011 9718341 PMC1377399

[B4] Bunker C. H. Berson E. L. Bromley W. C. Hayes R. P. Roderick T. H. (1984). Prevalence of Retinitis Pigmentosa in Maine. Am. J. Ophthalmol. 97 (3), 357–365. 10.1016/0002-9394(84)90636-6 6702974

[B5] Chakraborty R. Kimmel M. Stivers D. N. Davison L. J. Deka R. (1997). Relative Mutation Rates at Di-, Tri-, and Tetranucleotide Microsatellite Loci. Proc. Natl. Acad. Sci. 94 (3), 1041–1046. 10.1073/pnas.94.3.1041 9023379 PMC19636

[B6] Chu Q. Martinez T. F. Novak S. W. Donaldson C. J. Tan D. Vaughan J. M. (2019). Regulation of the ER Stress Response by a Mitochondrial Microprotein. Nat. Commun. 10 (1), 4883. 10.1038/s41467-019-12816-z 31653868 PMC6814811

[B7] Colombo R. (2000). Age and Origin of the PRNP E200K Mutation Causing Familial Creutzfeldt-Jacob Disease in Libyan Jews. Am. J. Hum. Genet. 67 (2), 528–531. 10.1086/303021 10889050 PMC1287202

[B8] Daiger S. P. Sullivan L. S. Bowne S. J. Rossiter B. J. F. (2021). RetNet Retinal Information Network. University of Texas-Houston Health Science Center.

[B9] Eckert K. A. Hile S. E. (2009). Every Microsatellite Is Different: Intrinsic DNA Features Dictate Mutagenesis of Common Microsatellites Present in the Human Genome. Mol. Carcinog. 48 (4), 379–388. 10.1002/mc.20499 19306292 PMC2731485

[B10] Gandolfo L. C. Bahlo M. Speed T. P. (2014). Dating Rare Mutations from Small Samples with Dense Marker Data. Genetics 197 (4), 1315–1327. 10.1534/genetics.114.164616 24879464 PMC4125402

[B11] Genomes Project C. Auton A. Brooks L. D. Durbin R. M. Garrison E. P. Kang H. M. (2015). A Global Reference for Human Genetic Variation. Nature 526 (7571), 68–74. 10.1038/nature15393 26432245 PMC4750478

[B12] Goldstein D. B. Reich D. E. Bradman N. Usher S. Seligsohn U. Peretz H. (1999). Age Estimates of Two Common Mutations Causing Factor XI Deficiency: Recent Genetic Drift Is Not Necessary for Elevated Disease Incidence Among Ashkenazi Jews. Am. J. Hum. Genet. 64 (4), 1071–1075. 10.1086/302313 10090892 PMC1377831

[B13] Hamamy H. Antonarakis S. E. Cavalli-Sforza L. L. Temtamy S. Romeo G. Ten Kate L. P. (2011). Consanguineous Marriages, Pearls and Perils: Geneva International Consanguinity Workshop Report. Genet. Med. 13 (9), 841–847. 10.1097/gim.0b013e318217477f 21555946

[B14] Hartong D. T. Berson E. L. Dryja T. P. (2006). Retinitis Pigmentosa. The Lancet 368 (9549), 1795–1809. 10.1016/s0140-6736(06)69740-7 17113430

[B15] Jia Y. Jucius T. J. Cook S. A. Ackerman S. L. (2015). Loss of Clcc1 Results in ER Stress, Misfolded Protein Accumulation, and Neurodegeneration. J. Neurosci. 35 (7), 3001–3009. 10.1523/jneurosci.3678-14.2015 25698737 PMC4331624

[B16] Jiao X. Li A. Jin Z. B. Wang X. Iannaccone A. Traboulsi E. I. (2017). Identification and Population History of CYP4V2 Mutations in Patients with Bietti Crystalline Corneoretinal Dystrophy. Eur. J. Hum. Genet. 10.1038/ejhg.2016.184 PMC5386409 28051075

[B17] Khan M. Azam M. Ajmal M. Collin R. den Hollander A. Cremers F. (2014). The Molecular Basis of Retinal Dystrophies in pakistan. Genes 5 (1), 176–195. 10.3390/genes5010176 24705292 PMC3978518

[B18] Li L. Chen Y. Jiao X. Jin C. Jiang D. Tanwar M. (2017). Homozygosity Mapping and Genetic Analysis of Autosomal Recessive Retinal Dystrophies in 144 Consanguineous Pakistani Families. Invest. Ophthalmol. Vis. Sci. 58 (4), 2218–2238. 10.1167/iovs.17-21424 28418496 PMC5397137

[B19] Li L. Jiao X. D’Atri I. Ono F. Nelson R. Chan C.-C. (2018). Mutation in the Intracellular Chloride Channel CLCC1 Associated with Autosomal Recessive Retinitis Pigmentosa. Plos Genet. 14 (8), e1007504. 10.1371/journal.pgen.1007504 30157172 PMC6133373

[B20] McElreavey K. Quintana-Murci L. (2005). A Population Genetics Perspective of the Indus Valley through Uniparentally-Inherited Markers. Ann. Hum. Biol. 32 (2), 154–162. 10.1080/03014460500076223 16096211

[B21] Nagasawa M. Kanzaki M. Iino Y. Morishita Y. Kojima I. (2001). Identification of a Novel Chloride Channel Expressed in the Endoplasmic Reticulum, Golgi Apparatus, and Nucleus. J. Biol. Chem. 276 (23), 20413–20418. 10.1074/jbc.m100366200 11279057

[B22] Narasimhan V. M. Patterson N. Moorjani P. Rohland N. Bernardos R. Mallick S. (2019). The Formation of Human Populations in South and Central Asia. Science 365 (6457). 10.1126/science.aat7487 PMC6822619 31488661

[B23] Reeve J. P. Rannala B. (2002). DMLE+: Bayesian Linkage Disequilibrium Gene Mapping. Bioinformatics 18 (6), 894–895. 10.1093/bioinformatics/18.6.894 12075030

[B24] Risch N. Leon D. d. Ozelius L. Kramer P. Almasy L. Singer B. (1995). Genetic Analysis of Idiopathic Torsion Dystonia in Ashkenazi Jews and Their Recent Descent from a Small Founder Population. Nat. Genet. 9 (2), 152–159. 10.1038/ng0295-152 7719342

[B25] Rivolta C. Sharon D. DeAngelis M. M. Dryja T. P. (2002). Retinitis Pigmentosa and Allied Diseases: Numerous Diseases, Genes, and Inheritance Patterns. Hum. Mol. Genet. 11 (10), 1219–1227. 10.1093/hmg/11.10.1219 12015282

[B26] Slatkin M. Rannala B. (2000). Estimating Allele Age. Annu. Rev. Genom. Hum. Genet. 1, 225–249. 10.1146/annurev.genom.1.1.225 11701630

[B27] Smith R. J. H. Holcomb J. D. Daiger S. P. Caskey C. T. Pelias M. Z. Alford B. R. (1989). Exclusion of Usher Syndrome Gene from Much of Chromosome 4. Cytogenet. Genome Res. 50, 102–106. 10.1159/000132733 2776474

[B28] World Population Prospects (2019). United_Nations_Department_of_Economic_and_Social_Affairs_Population_Dynamics. United Nations: United Nations; 2019 [cited 2021. Available at: https://population.un.org/wpp/ .

